# Anticancer, Cardio-Protective and Anti-Inflammatory Potential of Natural-Sources-Derived Phenolic Acids

**DOI:** 10.3390/molecules27217286

**Published:** 2022-10-26

**Authors:** Afnan, Ammara Saleem, Muhammad Furqan Akhtar, Ali Sharif, Bushra Akhtar, Rida Siddique, Ghulam Md Ashraf, Badrah S. Alghamdi, Saif A. Alharthy

**Affiliations:** 1 Department of Pharmacology, Faculty of Pharmaceutical Sciences, Government College University Faisalabad, Faisalabad 38000, Pakistan; 2 Riphah Institute of Pharmaceutical Sciences, Riphah International University, Lahore Campus, Lahore 38000, Pakistan; 3 Institute of Pharmacy, Faculty of Pharmaceutical and Allied Health Sciences, Lahore College for Women University, Lahore 54000, Pakistan; 4 Department of Pharmacy, University of Agriculture, Faisalabad 38000, Pakistan; 5 Department of Medical Laboratory Sciences, College of Health Sciences, University of Sharjah, University City, Sharjah 27272, United Arab Emirates; 6 Neuroscience Unit, Department of Physiology, Faculty of Medicine, King Abdulaziz University, Jeddah 22254, Saudi Arabia; 7 Pre-Clinical Research Unit, King Fahd Medical Research Center, King Abdulaziz University, Jeddah 22254, Saudi Arabia; 8 Department of Medical Laboratory Sciences, Faculty of Applied Medical Sciences, King Abdulaziz University, Jeddah 22254, Saudi Arabia; 9 Toxicology and Forensic Sciences Unit, King Fahd Medical Research Center, King Abdulaziz University, Jeddah 21589, Saudi Arabia; 10 Animal House Unit, King Fahd Medical Research Center, King Abdulaziz University, Jeddah 21589, Saudi Arabia

**Keywords:** phenolic acids, antioxidant, anti-carcinogenic, stroke, hypertension, anti-arthritis

## Abstract

Phenolic acids (PAs) are one of the utmost prevalent classes of plant-derived bioactive chemicals. They have a specific taste and odor, and are found in numerous medicinal and food plants, such as *Cynomorium coccineum* L., *Prunus domestica* (L.), and *Vitis vinifera* L. Their biosynthesis, physical and chemical characteristics and structure–activity relationship are well understood. These phytochemicals and their derivatives exert several bioactivities including but not limited to anticancer, cardioprotective, anti-inflammatory, immune-regulatory and anti-obesity properties. They are strong antioxidants because of hydroxyl groups which play pivotal role in their anticancer, anti-inflammatory and cardioprotective potential. They may play significant role in improving human health owing to anticarcinogenic, anti-arthritis, antihypertensive, anti-stroke, and anti-atherosclerosis activities, as several PAs have demonstrated biological activities against these disease during in vitro and in vivo studies. These PAs exhibited anticancer action by promoting apoptosis, targeting angiogenesis, and reducing abnormal cell growth, while anti-inflammatory activity was attributed to reducing proinflammatory cytokines. Pas exhibited anti-atherosclerotic activity via inhibition of platelets. Moreover, they also reduced cardiovascular complications such as myocardial infarction and stroke by activating Paraoxonase 1. The present review focuses on the plant sources, structure activity relationship, anticancer, anti-inflammatory and cardioprotective actions of PAs that is attributed to modulation of oxidative stress and signal transduction pathways, along with highlighting their mechanism of actions in disease conditions. Further, preclinical and clinical studies must be carried out to evaluate the mechanism of action and drug targets of PAs to understand their therapeutic actions and disease therapy in humans, respectively.

## 1. Introduction

Phytochemicals are not only a pivotal source of numerous active pharmaceuticals but also help in the adaptation of plants to their natural environment [1]. Plants are considered to be the primary source of naturally occurring bioactive compounds such as secondary metabolites and antioxidants. Approximately one to two hundred thousand plant secondary metabolites have been discovered in the world, of which almost 8000 naturally existing compounds belong to the class of phenolics [2,3]. Vegetables, fruits, cereals, and nuts are ample sources of polyphenols. 

Polyphenols are a diverse and structurally complex group, with molecular weights between 50 to 3000 Daltons. Polyphenols are further distributed into numerous major classes, including flavonoids, phenolic acids (PA), stilbenes, and lignans. Coumarins (simple and polycyclic) exist as a separate subclass. Flavonoids, as a major class, can be characterized as flavanones, isoflavones, flavanols, anthocyanins, and flavonolignans [4]. PAs are well-distributed in plants such as *Coffea arabica* L., *Camellia sinensis* (L.), *Cynomorium coccineum* L., and *Phyllanthus embelica* L. The second most common subclasses of polyphenols are chiefly categorized into benzoic and cinnamic acid derivatives [3,5]. Another class, “stilbenes”, is only produced during pathogenesis. Lastly, lignans are known as phytoestrogens with a high abundance in flaxseed oil. Generally, the structural features of the polyphenol family contribute substantially to its bioavailability, pharmacokinetics, biomolecular interactions and effectiveness [4]. 

In contrast to flavonoids, free PAs, for instance, hydroxybenzoic acid (HBA) and hydroxycinnamic acid (HCA), have profound water solubility and bioavailability. Similar to flavonoids, they have profound antioxidant action. PAs and flavonoids have gained more interest because of their potential biological actions, such as antioxidant, cardioprotective, anti-inflammatory, anti-atherosclerotic, immunoregulatory, anti-allergenic, anti-thrombolytic, antimicrobial, antitumor, anti-obesity, anticancer and anti-diabetic properties [6,7,8]. This study aims to provide information on the distribution of phenolic acids in natural plants in the rich quantities required for their potential pharmacological activities, and their mechanisms of action in disease prevention. From the literature review, the use of PAs as drugs is promising. However, more clinical research is required before this class of phytochemicals can be used for treatment. 

## 2. Sources and Classification of PAs

**Figure 1:**
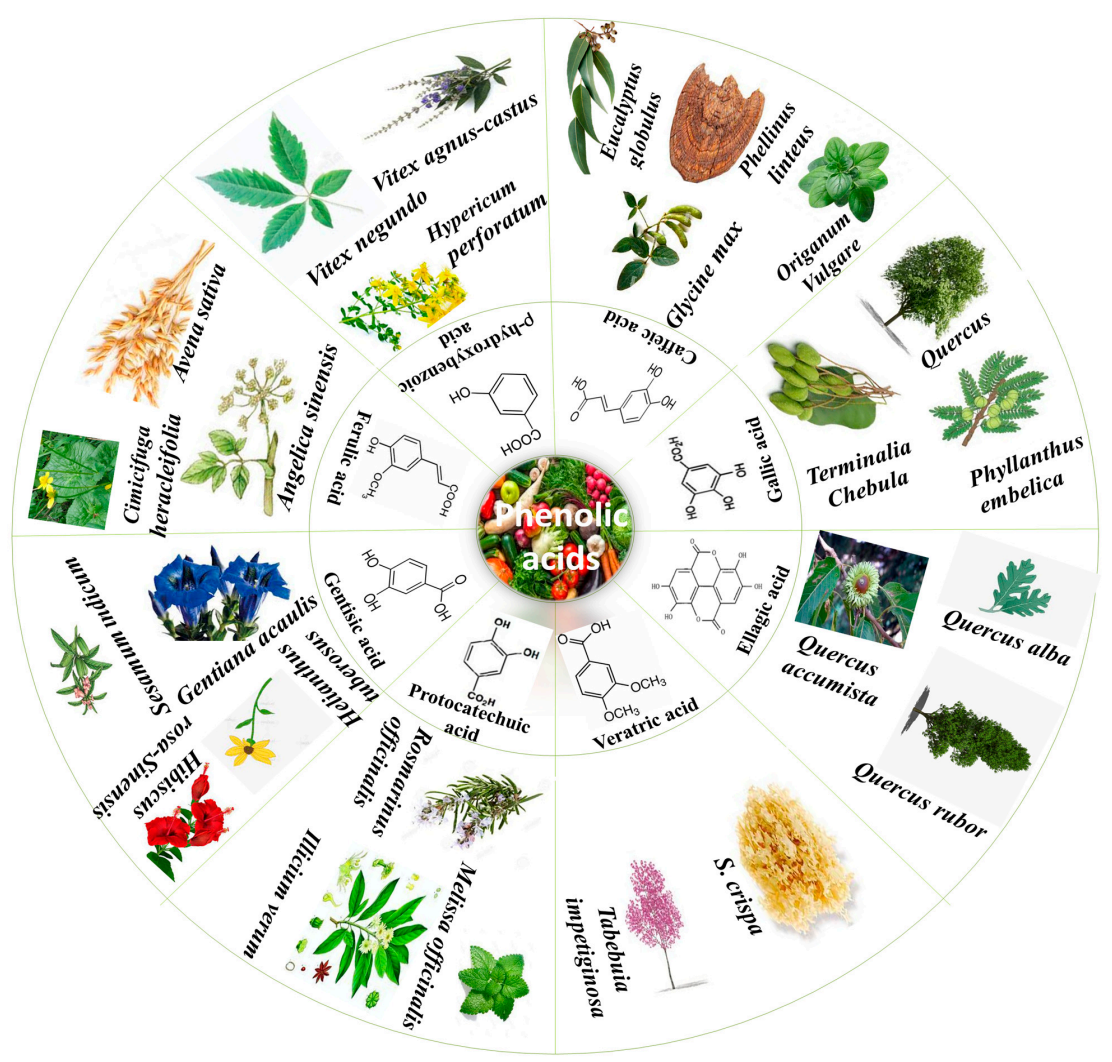
Phenolic acids and their plant sources.

**Figure 2:**
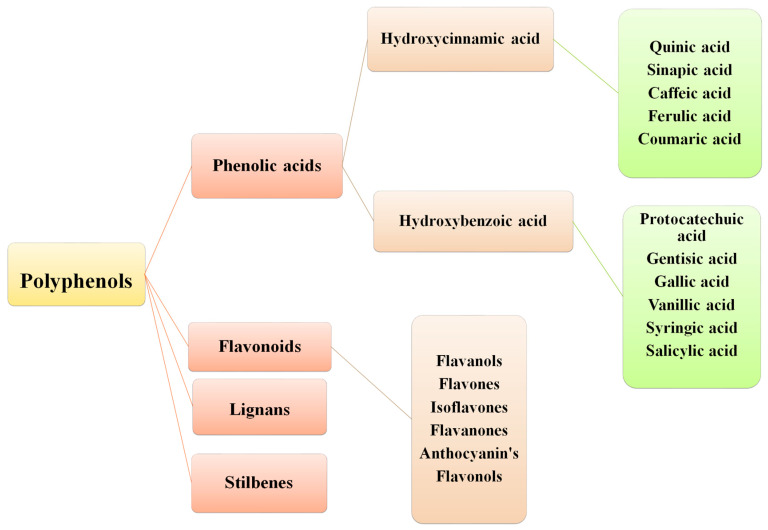
Classification of polyphenols.

PAs are obtained from all plant-derived food sources and different plant parts; for instance, seeds, stem, leaves and roots [9]. There is an unequal PA distribution throughout plants that depends on various factors such as stress, temperature and abiotic conditions [10]. Figure 1 summarizes some important plants from which various PAs in higher content have been isolated. The two main sub-classes of PAs are comprised of two distinguishing constitutive carbon substructures: hydroxybenzoic acid (HBA) and hydroxycinnamic acid (HCA), as described in Figure 2. 

## 3. Hydroxybenzoic Acid Derivatives and Their Sources

**Figure 3:**
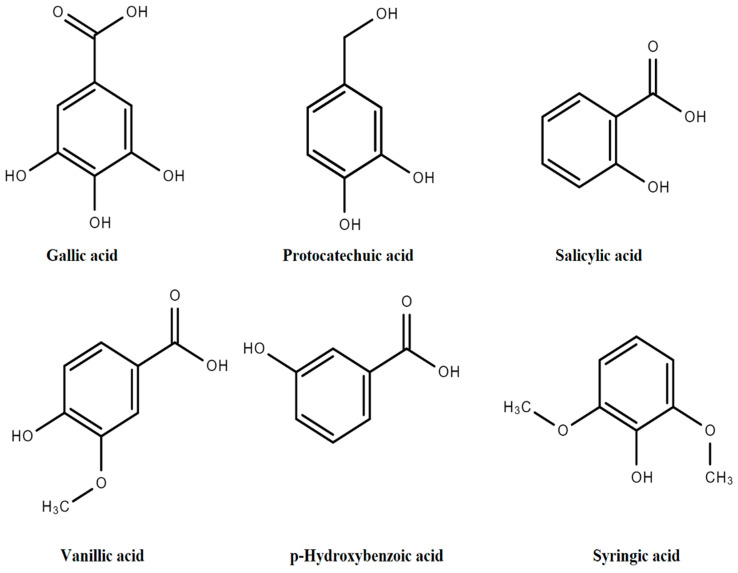
Derivatives of hydroxybenzoic acids.

The general structure of PAs includes C6-C1 rings from derivatives of benzoic acid (BA) such as *p*-hydroxybenzoic acid (*p*-HBA), salicylic acid (SA), 2,3-dihydroxybenzoic acid (2,3-DHBA), 3,4-dihydroxybenzoic acid (3,4-DHBA), 2,5-dihydroxybenzoic acid (2,5-DHBA), 3,5-dihydroxybenzoic acid (3,5-DHBA), gallic acid (GA), vanillic acid (VA), and syringic acid (SyA), as presented in Figure 3 [11]. 

A variation in structures arises due to hydroxylation and methylation of the aromatic ring [3,12]. Derivatives of HBAs are mostly obtained from vegetables. Other derivatives such as SA, GA and VA are obtained in small quantities from *Valerianella locusta* L., *Foeniculum vulgare* Mill., *Petroselinum crispum* (Mill.), and *Spinacia oleracea* L. *Rheum rhabarbarum* L. contains a minor concentration of GA, followed by protocatechuic acid (PRCA) and VA. *Allium cepa* L. has the highest concentrations of PRCA in outer dry skin and a minor proportion is accumulated in pulp tissues [13]. 

*Daucus carota* L., *Apium graveolens* L. and *Armoracia rusticana* G., B.Mey and Scherb contain small quantities of *p*-HBA, GA, SyA and VA. Gentisic acid is found in small amounts in vegetables and crops such as *Solanum lycopersicum* L., *Solanum melongena* L., *Capsicum* species, *Cucumis melo* L. and *Cucumis sativus* L. *Brassica oleracea* L. contains minor quantities of *p*-HBA, GA, gentisic acid, and ferulic acid (FA) [8].

## 4. Derivatives of Hydroxycinnamic Acids and Their Sources

**Figure 4:**
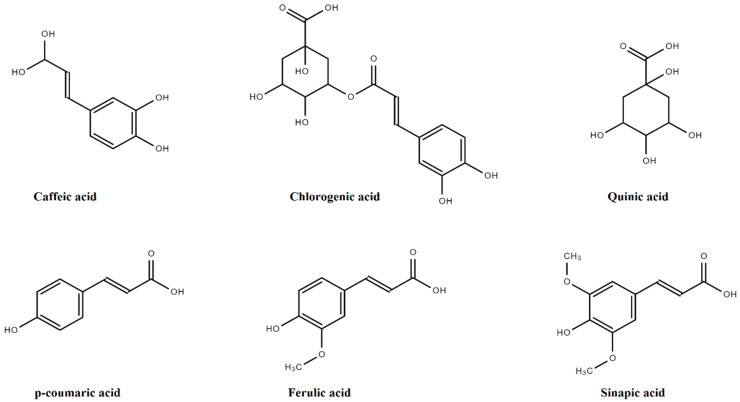
Derivatives of hydroxycinnamic acids.

Quinic acid (QA) is the basic derivative of HCA, having four hydroxyls plus one carboxylic acid moiety. Sinapic acid (SA), *p*-coumaric acid (PCA), FA, and caffeic acids (CA) are amongst the major derivatives of HCA that originate in the free form, while glycosylated derivatives of QA, tartaric acid (TA), and shikimic acid (ShA) are the bound forms, as presented in Figure 4. 

**Figure 5:**
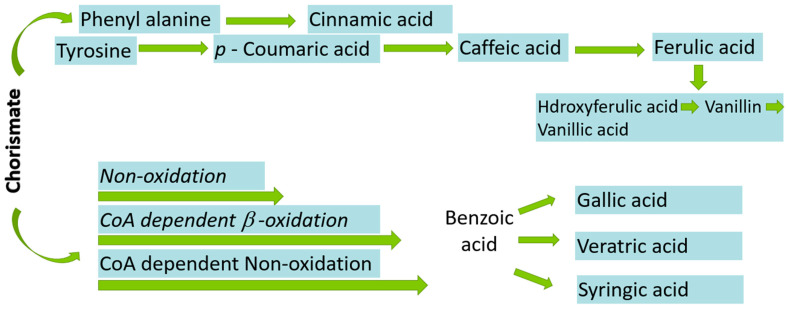
Biosynthesis of phenolic acids from chorismate.

Chlorogenic acid, formed by a combination of QA and CA, is present in several fruits such as *Vaccinium myrtillus* L., *Prunus domestica* L., *Prunus avium* L., and *Malus domestica* (Suckow) Borkh. These have 0.5–2 g HCA/kg. In *Solanum tuberosum* L., chlorogenic acid is the chief PA, ranging from 0.20–2193.0 mg/100 g of dry weight, as mentioned in Figure 5 [14]. 

HCA is mainly found in leafy vegetables such as *Brassica* species, *Lactuca sativa* L., and *Amaranthus cruentus* L., etc. HCA is found in high levels in ripened fruits in contrast to other parts of the plant. However, their concentrations usually reduce during ripening. High amounts of synaptic acids are found in *Nasturtium officinale* W.T., *Lepidium sativum* L., *Brassica rapa* L., and *Brassica juncea* (L.) [15]. The FA (0.8–2 g/kg of dry weight) is among the most abundant HCA that is present in cereals and wheat grains as esterified to arabinoxylans [16]. The FA is also found in *Brassica* species, *Abelmoschus esculentus* (L.), *Vigna unguiculate* (L.), and *Portulaca oleracea* (L.) [17]. Generally, CA, GA, FA and PCA contents in *Solanum tuberosum* L. are about 5 mg/100 g dry weight [8].

## 5. Biosynthesis of PAs

L-phenylalanine, or L-tyrosine, is the primary precursor in the ShA pathway for the synthesis of Pas in bacteria, fungi and plants. The mevalonic acid pathway is also involved in the synthesis of PAs in higher plants but is of less importance. A total of seven enzymatic phases lead to a last-known precursor called chorismite, as mentioned in Figure 5 [18]. 

Firstly, phosphoenolpyruvic acid (PEP) is produced via the glycolytic pathway. The carbohydrate (non-oxidative) D-erythrose-4-phosphate (E4P) is then condensed via aldol condensation with PEP to yield 3-deoxy-Darabino-heptulosonic acid 7-phosphate (DAHP). In the second step, cyclization of DAHP yields 3-dehydroquinic acid (DHQ). In the next step, dehydration of DHQ leads to formation of 3-dehydroshikimic acid (DHS). Then, shikimate (ShA) is activated to ShA 3-phosphate (S3P), followed by condensation to chorismic acid that further converts into various PAs such as CA, PCA, BA, CA and FA as shown in Figure 5, while DHS leads to formation of GA and EA [19,20]. 

## 6. Structure Activity Relationship of PAs

**Figure 6:**
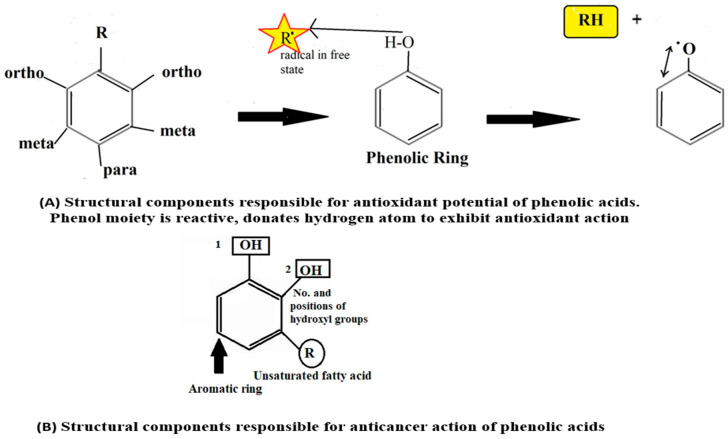
Structural activity relationship of phenolic acids. 1 and 2 showed hydroxyl groups.

The pharmacological activities, such as antioxidant, anti-inflammatory, anticancer, etc., of Pas depend upon its structural components, such as number and position of hydroxyl groups (-OH), unsaturated fatty acid chain, methoxy (-OCH_3_), and carboxylic acid (-COOH), as described in Figure 6A. The number of hydroxyl groups is proportional to antioxidant activity [21].

The anti-cancer properties of PAs vary from one compound to another based upon their structural variability and molecular targets. The number and position of phenolic hydroxyl groups also impart crucial roles in the anticancer action of PAs (Figure 6B). There is a direct relationship between the -OH group and anticancer potential. For instance, PAs are devoid of anticancer action with the absence of an -OH group and the presence of methoxy (OCH3) group. Additionally, the existence of unsaturated short-side chains of fatty acids also contributes to the activity [12].

## 7. Pharmacological Activities of PAs

**Figure 7:**
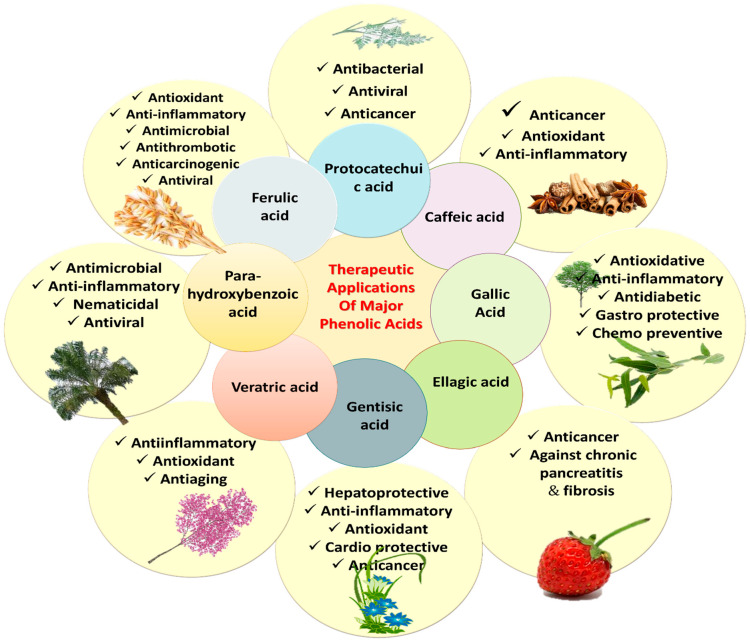
Therapeutic uses of major phenolic acids.

The literature survey validates the vital role of PAs in the treatment of various diseases, prophylaxis and other health benefits. Recent updates have linked antioxidant properties of PAs with the prevention of diseases such as cardiopulmonary and cardio-metabolic diseases, stroke, myocardial infarction (MI), and cancers [22]. Various PAs, such as CA, ellagic acid (EA), ShA, PCA, gentisic acid, VA, SA, PCA, FA, and 3,4-DHBA have exhibited anticancer, antiapoptotic, hepatoprotective, antidiabetic, nephroprotective, anti-inflammatory, analgesic, antioxidant, gastroprotective and cardioprotective potential in numerous in vitro and in vivo studies (Table 1). The *p*-hydroxybenzoic acid (*p*-HBA) is known for anti-inflammatory and antimicrobial potential. The PCA is known for antibacterial, antiviral and anticancer activities. VA is effective for its anti-inflammatory, antiaging and antioxidant action. Various PAs and their bioactivities are shown in Figure 7. 

### 7.1. Anti-Cancer Actions of PAs

The “two-steps carcinogenesis” hypothesis elaborates how a single cell is generated from a normal cell, which leads to the progression of cancer [42]. PAs exhibit anti-cancer action by promoting apoptosis, targeting angiogenesis, and reducing abnormal cell growth that might be due to an aromatic ring, location of hydroxyl groups and highly unsaturated chains in their structure [43].

**Figure 8:**
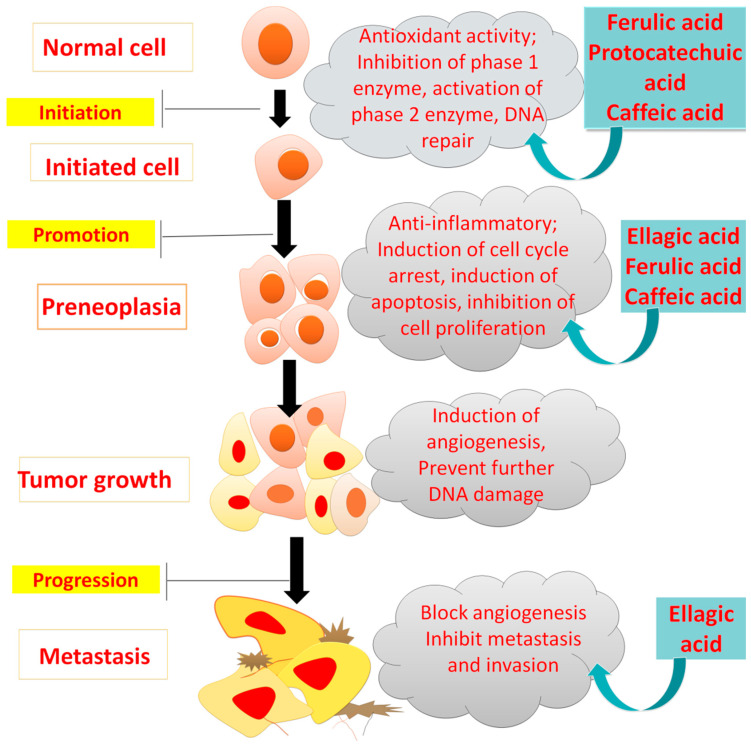
Inhibitory effects of phenolic acid on cancer development.

Mutations in phosphatidyl inositide 3-kinase (PI3Ks or Akt) molecules and mitogen-activated protein kinases (MAPKs), or the Ras/Raf/Erk pathway, are the crucial steps for most cancer types blocked by PAs as described in Figure 8 [44,45]. 

Epidemiological studies have shown the role of PA-rich fruits and vegetables in reducing several cancers, suggesting their efficacy against cancer incidence, prevention and mortality [7]. Most PAs inhibit different cancers depending on their antioxidant capacity, and regulation of transcriptional factors, such as nuclear factor–erythroid-related factor (Nrf-2) [46,47,48]. The studies on the anticancer action of various PAs are described in Table 2. 

#### 7.1.1. Anticancer Activity of EA

It was revealed that the EA had induced apoptosis and inhibited cell cycle and cell growth in cervical squamous carcinoma cells (CaSki) time- and concentration-dependently. Within two days of the cycle, EA arrested G1 phase dose dependently, representing the sensitivity of these cells towards EA [49]. 

NF-κB has potential activity against inflammation and cancers. However, its role in cancer progression is multifarious. Two transcription factors, p50 and p65, in the absence of any stimulus, are sequestered in a regulated signal transduction regulator (I-κBα) activated by the I-κBα kinase enzyme. This causes proteasome deprivation of I-κBα [48]. NF-κB controls the expression of the proteins controlling the cell production, existence, angiogenesis, and metastasis. Signal transducer and activator of transcription (STAT-1) causes up-regulation of cells that ends in cancer cell survival [50]. On the contrary, down-regulation of NF-κB causes cell sensitization and eventually apoptotic action. Genes such as Bcl-2 or XL, survivin, Cyclin D1, TRAF1 and 2 block apoptosis by up-regulation of NF-κB. The apoptosis is activated by the activation of caspase-9, -3, -8, and -7, which is hindered by the loss of p53 protein (tumor suppressant). The EA can delay cell development and cell cycle movement by initiating apoptosis of prostate, colorectal, oral, pancreatic, and bladder cells [51].

#### 7.1.2. Anticancer Activity of FA and PCA

A previous study reported that FA and PCA at 15 to 1500 µM blocked the G1, G2 and S phases in the Caco-2 cell line, demonstrating their anticancer action [52,53,54,55,56,57,58,59]. Gao et al. used the HCT116 cell line and reported that 3, 4-DHBA reduced proliferative activity, suggesting its use for prostate cancer treatment [60,61,62]. Previously, SyA exhibited anticancer potential by restraining cell proliferation and reducing oxidative stress and NF-κB expression. The most notable action was observed at 1200 µM on Human SW-480 cells [63].

Bouzaiene et al. repoted the antioxidant and anticancer effects of CA, FA and PCA on superoxide production and cell proliferation using colon adenocarcinoma (HT-29-D4) and human lung cell lines (A549). It was found that the highest concentration of 200 μM of all test compounds had inhibited cell migration and superoxide anoin production [53]. Previously, Janicke et al. reported that FA and PCA had reduced the cell proliferation and cell cycle phase distribution at 1500 μM in Caco-2 cells [52]. 

#### 7.1.3. Anticancer Activity of CA and Derivatives

CA has also demonstrated significant anticancer potential. A study on CA revealed its anti-proliferative, free radical scavenging activity, mitochondrial colony development and apoptotic effects in HCT15 cells with an IC_50_ of 800 μM [54]. In another study, it was reported that the different derivatives of CA inhibited cell proliferation in vitro in a dose-dependent manner. The IC_50_ for CA phenethyl ester (CAPE) and CA phenyl propyl ester (CAPPE) in the CRC HCT-116 cell line were 44.2 and 32.7 mM, respectively, while in human CRC SW-480 cells, IC_50_ were 132.3 mM and 130.7 mM, respectively. These derivatives significantly augmented the signaling pathways and also induced G0/G1 cell cycle arrest. In an animal model, CAPE- or CAPPE-mediated suppression of tumor growth was related to the modulation of the PI3-K/Akt, AMPK and mammalian target of rapamycin (mTOR) signaling pathways in experimental animals as described in Table 2 [55]. 

### 7.2. Anti-Inflammatory and Analgesic Actions of PAs

PAs are known for their anti-inflammatory and anti-nociceptive activities during arthritis, headache, rheumatoid arthritis, neuropathic pain, backache, etc. ShA at 100 and 200 mg/kg exhibited analgesic action in dopamine, inducing mechanical hyperalgesia in mice via decreasing tumor necrosis factor (TNF-α), nitrite production, and interleukin (IL)-1β [64,65,66,67,68,69,70,71].

**Figure 9:**
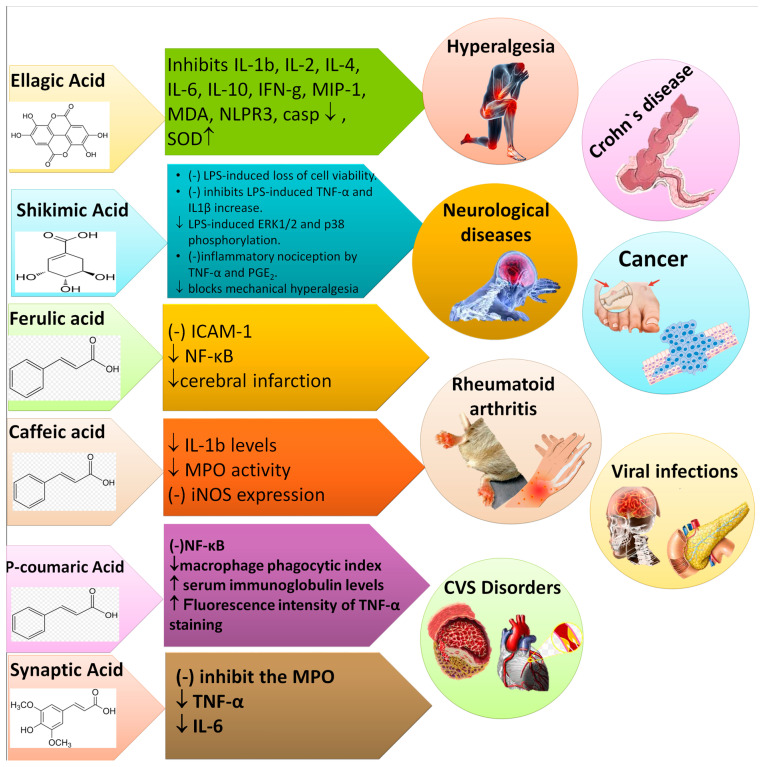
Phenolic acids and their target biomarkers in different inflammatory diseases. ↓ showed decreased, while ↑ showed increased.

FA has shown anti-inflammatory activity in several models of disease. FA at 100 mg/kg reduced cerebral infarction in middle cerebral artery occlusion (MCAO) and exhibited anti-inflammatory effect in rats [56]. Meanwhile, the anti-inflammatory activity of PRCA was observed at 1–25 μM in LPS-induced inflammation in RAW 264.7 cells via reducing TNF-α, IL-1β, and prostaglandin (PGE2) [57]. The mechanisms of action and effect of different PAs on various inflammatory diseases are explained in Figure 9.

PCA and SA have demonstrated anti-inflammatory activity through modulation inflammatory cytokines. PCA at 100 mg/kg exhibited anti-arthritic and immunosuppressant action in Complete Freund’s Adjuvant (CFA)-induced arthritic rats via reducing NF-κβ, and macrophage phagocytic index [72,73]. SA at 819–2500 mg/kg dose exhibited anti-inflammatory activity in male mice via downregulation of TNF-α and IL-6 [58]. Various research has reported the anti-inflammatory and analgesic activities of PAs, as described in Table 3.

**Table 2:** Anticancer activity of phenolic acids.

Phenolic Acids	Cell Line/Animal Model	Dose	Mechanism	Type of Cancer	References
Caffeic acid, coumaric acid, ferulic acid	HT-29-D4 A549	50,100, 150 and 200 µM for 1 day	(-) cell proliferation (-) ROS production (-) cell adhesion	Lung carcinoma and colon adenocarcinoma	[53]
Ferulic acid, *p*-coumaric acid	Caco-2	15, 150 and 1500 µM for 3 days	↓ cell growth ↓ cell proportion in G1 phase	Colon cancer	[52]
Caffeic acid	HCT15	500, 1000 and 2000 µM	(-) cell viability (-) colony development ↑cell accumulation at G1 ↑ scavenging activity √ apoptosis	Colon cancer	[59]
3,4-Dihydroxyphenylacetic acid	HCT116	600 mg for >1.5 days	↓ proliferative activity	Prostate and colon cancer	[60]
Cycloartenyl ferulate	SW480 SW620 Colo-201	40, 80 and 160 mM for 3 days	√ apoptosis in metastatic cancer cells √ tumor regression (-) cell growth	Colorectal adenocarcinoma	[61]
	Male nude mice	1.6 and 32 mg/kg for 10 days			
Dicaffeoylquinic acid	DLD-1	10, 100, 500 and 1000 µM for > 3 days	(-) cell growth and mutation √ apoptosis	Colon cancer	[42,62]
CAPE and CAPPE	BALB/C mice	50 nmol/kg/day for 42 days	↓ colorectal tumor (-) PCNA, FASN and MMP ↑Cell cycle arrest (-) cell growth by controlling P13K/AkT and mTOR cascades	Colon cancer	[55]
	HCT-116SW-480	0, 5, 10, 20, 50 and 100 µM IC50s in CRC HCT-116 for CAPE 44.2 mM and CAPPE 32.7 mM In SW-480			
Syringic acid	SW-480	(2000 μM)	(-) cell proliferation ↑apoptosis Down-regulated NF-κB Modulate oxidative stress	Colorectal cancer	[63]
	Rats	50 mg/kg for 210 days			
Ellagic acid	MCF-7 cells	10^−7^ to 10^−9^ M	↑Estrogenic activity of ER-α, β	Breast cancer	[64]
Ellagic acid	SiHa, C33A and HeLa cells	30 μM	(-) cell propagation Arrest G1 √Apoptosis (-) phosphorylation of JAK2 signaling and STAT3 expression	Cervical carcinoma	[65]
Ellagic acid	ES-2 PA-1 MRC-5	10–100 μM	G1 arrest ↑ P53 and p21 ↓ cyclin D1 and E.	Ovarian cancer	[51]
Ellagic acid	AIH (PC-3) and PLS10	80–200 μM	(-) invasive potential through action on the activity of proteases, such as collagenase/gelatinase and collagenase IV	Prostate cancer	[66]
3-O-Methylgallic acid and gallic acid	Caco-2	10–100 mM	↓ cell viability, S-phase √ apoptosis (-) G0/G1 phase, NF-κβ, AP-1, STAT-1, OCT-1	Colon cancer	[50]
Gallic acid	22Rv1 and DU145, PWR-1E	0–100 µM	(-) tumor cell proliferation (√) apoptosis ↓ micro vessel density in tumor	Prostate cancer	[67]
	Athymic male nude mice	CG or 0.3 and 1% (*w*/*v*) dose of GA for 5 days/week			
Vanillic acid	male Balb nude mice	30 mg/kg	(-) HIF-1α by suppression of mTOR/p70S6K/4E-BP1 Raf kinase/MEK pathways and angiogenesis	Prostate cancer	[68]
	HCT116, Hep3B A549 HUVEC cells	0–30 µM			
Sinapic acid	PC-3 LNCaP	250–4000 µM	(-) cell proliferation ↓ caspase-3 activity (-) cell invasion ↑ expression of BAX ↓ MMP-9 in PC-3 cell expression In LNCaP cells, ↑ BAX, CASP3, CASP7 and CYCS ↓ CDH2, MMP-2 and MMP-9 cells expression	Prostate cancer	[69]

(-) inhibition, suppression; (√) induction, (↓) decrease/reduction; (↑) increase; colon adenocarcinoma (HT-29-D4), human lung cell line (A549), human colorectal carcinoma (HCT), caffeic acid, phenethyl ester (CAPE), phenyl propyl ester (CAPPE), proliferating cell nuclear antigen (PCNA), fatty acid synthase (FASN), matrix metalloproteinases (MMP), Phosphatidylinositol-3-kinase (P13K/Akt), mammalian target of rapamycin (mTOR).

**Table 3:** Anti-inflammatory and analgesic actions of phenolic acids.

Phenolic Acid/Source	Animal Model/Cell Line	Dose	Mechanism of Action	Result	References
Brazilian green propolis extract (EPP-AF)	LPS-induced macrophages in Swiss mice	30, 100 and 300 μg/mL for 18 h	↓ IL-1β, casp-1↓ (-) inflammosome activation	Regulates inflammasome path and prevents inflammatory activity	[70]
Shikimic acid	Dopamine inducing mechanical hyperalgesia in mice	100 and 200 mg/kg for 30–180 min	↓ cell viability ↓ TNF-α, nitrite production, IL-1β	Prevents inflammation and mild to moderate pain	[71]
Ferulic acid	Middle cerebral artery occlusion (MCAo) in male rats	(100 mg/kg i.v.) for 1 day	(-) ICAM-1 ↓ NF-κB	Reduces cerebral infarction and possess anti-inflammatory effect	[56]
Caffeic acid Butyl and octyl esters	Carrageenan-induced paw edema in mice	30 mg/kg for 5 days	↓ IL-1β levels ↓ MPO activity ↑ iNOS	Anti-inflammatory action	[72]
*p*-coumaric acid	CFA induces arthritis in rats	100 mg/kg	(-) NF-κB ↓ macrophage phagocytic index ↑the serum immunoglobulin	Immunosuppressant and anti-inflammatory agent in arthritis	[73]
Sinapic acid	TPA and AA induced ear edema in mice	0, 819, 1024, 1280, 1600, 2000, 2500 mg/kg for two weeks	C inhibit the MPO ↓ TNF-α ↓ IL-6	Inhibits acute and chronic inflammation	[58]
Shikimic acid	Macrophage (RAW 264.7) cell DMEM	10 mM	(-) cell viability and nitrite accumulation ↓ TNF-α, IL-1β	Inhibited LPS-induced cellular pro-inflammatory cytokines	[71]
Caffeic acid derivatives	LPS stimulated RAW 264.7, DMEM	0.5 mg/ml	↓ Nitrite accumulation (-) iNOS expression	In vitro anti-inflammatory action	[72]
Protocatechuic acid	RAW 264.7 cells, RPMI 1640	1, 2, 5, and 25 μM	↓ TNF-α, IL-1β, NO and PGE_2_	↓ TNF-α and IL-1β ↓ NO and PGE_2_ (-) iNOS and COX-2 deprivation (-) phosphorylated NF-κβ, p38, ERK, and JNK	[57]
Protocatechuic acid	MAEC, RPMI 1640	0.05, 0.5, 5.0, 10, 20, and 40 μmol/L	(-) adhesion of HL-60 cells to MAEC’s (-) VCAM-1 and ICAM-1 mRNA expression↓ NF-κβ initiation ↓ TNF-α-induced cellular damage and monocyte adhesion	Anti-inflammatory	[74]

(-) inhibition/suppression; (√) induction, (↓) decrease/reduction; (↑) increase; 12-O-tetradecanoylphorbol-acetate (TPA); arachidonic acid (AA).

### 7.3. Cardio-Protective Actions of PAs

PAs have been widely studied for their protective effects against cardiovascular disorders such as atherosclerosis, ischemia, stroke and hypertension. Cardiopulmonary disorders are among the foremost causes of deaths in both economically developed and developing states [75,76,77,78,79,80,81,82,83,84,85,86,87]. It was found that CAPE derivatives of caffeic acid at 3 and 15 mg/kg had shown cardio-protective activity against I/R injury in rabbits. The results suggested an increased inhibition of MPAK phosphorylation along with a decline in IL-1B and TNF-a expression [88,89,90,91,92]. In another study, it was revealed that syringic acid and revasterol at 50 mg/kg had shown efficient cardio-protective action in cardiotoxicity induced by isoproterenol for 30 days in rats [92].

#### 7.3.1. Atherosclerosis

**Figure 10:**
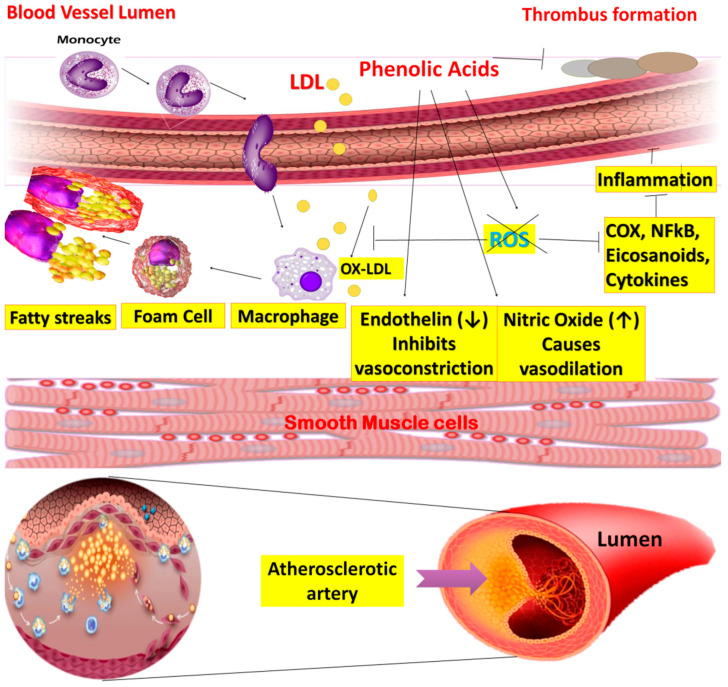
Mechanism of anti-atherosclerosis effect of phenolic acids.

Atherosclerosis, a chronic inflammatory disease, is characterized by the deposition of white blood cells in the vessels. Risk factors contributing to the development of the disease include high blood cholesterol levels, specifically low-density lipoproteins (LDL). Platelets bind with leukocytes via P-Selectin or P-Selectin glycoprotein ligand-1 (PSGL-1) to form thrombus and atherosclerotic depositions in the walls [76]. The anti-atherosclerotic mechanism of PA has been presented in Figure 10.

In atherogenesis, the key factors are endothelial dysfunction and oxidative modification of LDL. Normally, endothelial cells maintain the integral structure of vessels and act as a permeable wall. Moreover, it is also responsible for regulating vascular tone by producing excess nitric oxide (NO), growth hormones, and prostaglandins [77].

PAs such as GA exhibits anti-atherosclerotic activity because of its association with white blood cells and inhibition of platelets. P-selectin endothelial expression is activated by a decrease in intracellular calcium. This stimulation occurs by adenosine diphosphate (ADP) via controlled signal transduction of PKC*α*/p38 MAPK and Akt/GSK3*β* [78]. The studies on cardioprotective action of various PAs are described in Table 4. 

A previous study showed that protocatechuic aldehyde, a derivative of protocatechuic acid derived from *Salvia miltiorrhiza*, had shown reduced platelet-derived growth factor (PDGF)-induced growth and movement of smooth muscle cells through regulation of the (PI3K)/Akt and M Kinase pathways ([79]).

It is previously found that EA proficiently diminished oxidative stress and plasma lipids, and prevented lipid peroxidation. It also blocked the oxidized LDL uptake in murine macrophages by down-regulating SR-B1 (a membrane receptor that controls the internalization of oxidized LDL, which eventually stimulates the deposition of cholesterol in cells). Likewise, the results also revealed EA induced ATP-binding cassette transporter (ABCA1) membrane receptor expression and cholesterol efflux in lipid-loaded macrophages. This ABCA1 regulates cholesterol homeostasis to protect an atherosclerotic event [80]. 

#### 7.3.2. Myocardial Infarction and Stroke

It was found that SA tended to prevent complications related to cardiovascular diseases such as myocardial infarction and stroke [81]. The mechanism is to obstruct the platelet-inhibitory role and activate an enzyme called Paraoxonase 1 that in return defends the oxidation of serum lipids and reduces macrophage formation and treats atherosclerosis [82]. Gentisic acid showed anti-hypertrophic and anti-fibrotic effects during an in vivo study on mice at a subsequent dosage of 100 mg/kg/day for three weeks through substantial down-regulation of the sp1 and ERK1 and 2 pathways [83]. In an in vivo study on cardiac damage induced with doxorubicin (5 mg/kg), gentisic acid prevented cardiotoxicity by preventing cardiac myofibrillar and hyalinization necrosis in male BALB mice [84].

Earlier research stated that FA at 20 mg/kg and ascorbic acid at 80 mg/kg had synergistically reduced MI by neutralizing oxidative stress and restored CAT and SOD biomarkers, and reduced CPK and LDH levels in isoproterenol-induced myocardial infarction in rats [85]. PRCA provided cardio-protection against streptozotocin-induced Type 1 diabetic rats at 50 and 100 mg/kg. During 12 weeks of treatment with PCA, cardiac function and autonomic nervous system balance were significantly restored due to improved cardiac mitochondrial damage (Semaming, Kumfu, Pannangpetch, Chattipakorn, and Chattipakorn, 2014). 

PAs have tremendous cardio-protective action through retarding atherosclerosis via acting on thrombin-induced matrix invasion of vascular smooth muscle cells. They protect against angiotensin II-induced hypertension in rats by blunting endothelial dysfunction and promoting formation of nitric oxide [86]. Furthermore, PAs prevent the damage caused by ischemic reperfusion by activating pro-survival pathways [87].

#### 7.3.3. Hypertension

This is totally dependent on lifestyle and dietary modifications. Alterations in nitric oxide (NO), which controls blood pressure and myocardial injury, might be responsible for hypertension pathogenesis due to increased vasoconstriction. Vanillic acid has literature evidence of treating hypertension-induced cardiovascular complications [88]. The VA has normalized hypertension and left ventricular function by up-regulating mRNA expression of eNOS in L-NAME-induced hypertensive rats. Vanillic acid (VA) also dropped cardiac biomarkers, e.g., CK/CK-MB, and LDH. It also enhanced nitric oxide metabolite value in tissues, as described in Table 4 [76].

**Table 4:** Cardioprotective action of phenolic acids.

Phenolic Acids	Animal Model and Dose	Method	Biomarkers	Result	References
Gentisic acid	100 mg/kg/day for 3 weeks in mice	Cardiac hypertrophy and cardiac fibrosis induced by transverse aortic restriction	Down-regulation of Sp1 and ERK1 or 2 pathways	Anti-hypertrophic and anti-fibrotic effects	[83]
Gentisic acid	5, 10, 20 and 40 mg/kg/day for 28 days in Balb mice	Cardiac damage with doxorubicin	↓ cardiac myofibrillar necrosis, ↓ hyalinization necrosis ↓ cardiac toxicity	Prevented cardiotoxic effects of doxorubicin and treated cardiac damage	[84]
Caffeic acid phenyl ester (CAPE)	CAPE 1 h before (3 and 15 mg/kg) or 30 min after (15 mg/kg) the onset of ischemia in rabbits	Acute myocardial ischemia reperfusion injury	(-) p38 MPAK phosphorylation and caspase activation ↓ LDH, CK, CK-MB induced by I/R injury, ↓ IL-1β and TNF-α	Cardioprotective effects against I/R injury	[89]
Ferulic acid and ascorbic acid	FA = 20 mg/kg and AsA = 80 mg/kg for 6 days in rats	Isoproterenol-induced myocardial infarction in rats	Improved CAT, SOD↓ CPK and LDH levels	Synergistically reduced myocardial infarction and showed cardioprotective effects	[85]
Ferulic acid	20 mg and 40 mg/kg in Wistar rats	Doxorubicin-induced myocardial toxicity	(-) expression of ANP and BNP ↑myocardial GSH and Na+/K+ ATPase	Reduced cardiotoxicity	[90]
Syringic acid	H9c2 cardiomyocytes	hypoxia/reoxygenation injury	(-) apoptosis of cardiomyocytes ↓ p38 MAPK and JNK activation pathways	Inhibited apoptosis of cardiomyocytes, and prevented myocardial infarction	[91]
Syringic acid, revasterol	50 mg/kg for 30 days in rats	Isoproterenol-induced cardiotoxicity	↓ cardiac biomarkers, ↓ antioxidant enzymes ↓ docking with NF-κB and inflammatory markers	Cardioprotective	[92]
*p*-coumaric acid	8 mg/kg for 7 days in male rats	MI induced by isoproterenol	(-)cardiac hypertrophy and variations in lipoproteins (-)HMG-CoA reductase	Treated myocardial infarction and normalized the ECG	[93]
Protocatechuic acid (PCA)	250–500 mg/kg in male rats	MI/R injury	↓ Myocardial infarction, ↓ serum TNF-α ↓ platelets count	Up-regulation of phosphorylated Akt expression in cardiomyocytes and cardioprotective effects in rats	[94]
	Neonatal rat cardiomyocytes	Hypoxia, reoxygenation induced in cardiomyocytes	(-) apoptotic rate (-) cleaved caspase-		
Protocatechuic acid (PCA)	50 and 100 mg/kg for 84 days in male rats	T1DM induced by a streptozotocin	√cardiac function √ANS balance, (-) cardiac mitochondrial damage ↑ anti-apoptotic protein	Cardioprotective in type 1 diabetic rats	[95]

(-) inhibition/suppression; (√) induction, (↓) decrease/reduction; (↑) increase. Extracellular signal-regulated kinases (ERK), mitogen-activated protein kinase (MAPK), choline kinases (CK), choline kinase subunit (CK-MB), c-Jun N-terminal kinases (JNK), creatinine phosphokinase (CPK), atrial natriuretic peptide (ANP), brain natriuretic peptide (BNP), type-1 diabetes mellitus (T1DM).

## 8. Toxicity of Phenolic Acids

PA are known for antioxidant action because of the hydroxyl group, but they also act as pro-oxidants that lead to toxicity after reacting with redox-active metals. As a pro-oxidant, PAs deteriorate nucleic acid components, lipids and protein. The repeated intake of a high level of PAs is associated with allergic reactions and toxic effects. PAs are also known for tumorgenicity. At low doses, PAs such as GA, CIA, CA, and FA initiated cancer via stimulating Nrf-2 (redox regulator). There is an immense need to use PAs in the recommended dosage for the required duration in order to avail the therapeutic response, otherwise their use would be counterproductive [12].

## 9. Conclusions and Future Aspects

The association between phytochemicals and disease therapy is a major focus of health research. As reviewed, phenolic acids, either hydroxyl benzoic acids or hydroxycinnamic acids and their derivatives, have demonstrated significant potential for treatment and prevention of anticancer, anti-inflammatory and cardiovascular diseases. These activities of PAs have mostly been attributed to modulation of oxidative stress and signal transduction pathways.

However, further in vitro and in vivo studies regarding the efficacy, possible side effects, molecular mechanisms, and targets involved in specified physiological functions and pathologies, as well as clinical trials as adjuvants to already-used therapies, will explore further attributes of these phytochemicals, which will help to produce innovative pharmacological and nutraceutical products. These studies will guide in the drug development of safe and effective treatments of anticancer, inflammatory and cardiovascular diseases. As the clinical data lack a direction, we emphasize the evaluation of PAs in isolated or mixed forms for medicinal purposes. Moreover, targeted drug delivery approaches such as nano-formulation should be incorporated to achieve maximum beneficial effects and prevent toxicity. 

## Figures and Tables

**Table 1:** Phenolic acids and their therapeutic uses.

Phenolic Acid Class	Compound	Rich Plant/Fruit Source	Family	Part Used	Therapeutic Uses	Reference
	Di-gallic acid	*Acacia nilotica* (L.) *P.J.H. Hurter* and *Mabb.*	Fabaceae	Pods	Anti-inflammatory, antioxidant	[23,24]
	Ellagic acid	*Fragaria x ananassa (Duchesne ex Westson) Duchesne ex Rozier*	Rosaceae	Fruits	Anti-cancer, chronic pancreatitis, fibrosis	[8,25,26,27,28]
	Gallic acid	*Cynomorium coccineum* L. *Phyllanthus embelica* L.	Euphorbiaceae	Fruits	Anti-oxidant, anti-inflammatory, antiulcer, anti-diabetic	[21,29,30]
	Protocatechuic acid	*Prunus domestica* L. *Vitis vinifera* L.	Rosaceae Vitaceae	Fruit	Anti-oxidant, anti-inflammatory, antibacterial	[31]
Hydroxy cinnamic acids	Ferulic acid	*Avena sativa* L. *Oryaz sativa* L.	Gramineae	Seed	Anti-oxidant, anti-inflammatory, anti-viral, anti-microbial, anti-thrombotic, anti-carcinogenic	[32]
	*p*-HBA	*Elaeis guineensis Jacq.* *Pterocarpus santalinus* L.	Palmae Fabaceae	Dried leaves, stem, and bark	Antimicrobial, anti-mutagenic, anti-estrogenic, hypoglycemic, anti-inflammatory, nematicidal, antiviral	[33,34,35,36]
	Gentisic acid	*Gentiana acaulis* L.	Gentianaceae	Roots	Hepatoprotective, anti-cancer, anti-inflammatory, anti-oxidant, cardio-protective	[37,38,39]
	Caffeic acid	*Eucalyptus globulus Labill.*	Mitraceae	Wood, bark	Antioxidant, antimicrobial, anti-diabetic	[40,41]

*p*-HBA: *p*-hydroxybenzoic acid.

## Data Availability

Not applicable.

## References

[1] Rasouli H., Farzaei M.H., Khodarahmi R. (2017). Polyphenols and their benefits: A review. Int. J. Food Prop..

[2] Pereira D.M., Valentão P., Pereira J.A., Andrade P.B. (2009). Phenolics: From Chemistry to Biology. Molecules.

[3] Goleniowski M., Bonfill M., Cusido R., Ramawat K., Mérillon J.M. (2013). Phenolic Acids 63, in Phenolic Acids. Natural Products.

[4] Amawi H., Ashby J.C.R., Samuel T., Peraman R., Tiwari A.K. (2017). Polyphenolic Nutrients in Cancer Chemoprevention and Metastasis: Role of the Epithelial-to-Mesenchymal (EMT) Pathway. Nutrients.

[5] Abotaleb M., Liskova A., Kubatka P., Büsselberg D. (2020). Therapeutic potential of plant phenolic acids in the treatment of cancer. Biomolecules.

[6] Van Nies J.A., De Jong Z., van der Helm-van Mil A.H., Knevel R., Le Cessie S., Huizinga T.W. (2010). Improved treatment strategies reduce the increased mortality risk in early RA patients. Rheumatology.

[7] Adebo O.A., Gabriela Medina-Meza I. (2020). Impact of fermentation on the phenolic compounds and antioxidant activity of whole cereal grains: A mini review. Molecules.

[8] Rashmi H.B., Negi P.S. (2020). Phenolic acids from vegetables: A review on processing stability and health benefits. Food Res. Int..

[9] Macheix J.-J., Fleuriet A., Billot J. (2018). Fruit Phenolics.

[10] Sikorska M., Matławska I., Głowniak K., Zgórka G. (2000). Qualitative and quantitative analysis of phenolic acids in *Asclepias syriaca* L. Acta Pol. Pharm..

[11] Vuolo M.M., Lima V.S., Junior M.R.M. (2019). Phenolic compounds: Structure, classification, and antioxidant power. Bioactive Compounds.

[12] Anantharaju P.G., Gowda P.C., Vimalambike M.G., Madhunapantula S.V. (2016). An overview on the role of dietary phenolics for the treatment of cancers. Nutr. J..

[13] Mattila P., Hellström J., Törrönen R. (2006). Phenolic Acids in Berries, Fruits, and Beverages. J. Agric. Food Chem..

[14] Mohdaly A.A.A., Sarhan M.A., Mahmoud A., Ramadan M.F., Smetanska I. (2010). Antioxidant efficacy of potato peels and sugar beet pulp extracts in vegetable oils protection. Food Chem..

[15] Dos Santos Ferreira C.I., Pereyra A., Patriarca A.R., Mazzobre M.F., Polak T., Abram V., Buera M.d.P., Poklar Ulrih N. (2016). Phenolic compounds in extracts from Eucalyptus globulus leaves and Calendula officinalis flowers. J. Nat. Prod. Resour..

[16] El Gharras H. (2009). Polyphenols: Food sources, properties and applications—A review. Int. J. Food Sci. Technol..

[17] Alsulami A.L., Gull M. (2018). Screening of antimicrobial potential and bioactive components of selected medicinal plants against infectious bacterial isolates from leukemia patients. J. Exp. Biol. Agric. Sci..

[18] Heleno S.A., Martins A., Queiroz M.J.R., Ferreira I.C. (2015). Bioactivity of phenolic acids: Metabolites versus parent compounds: A review. Food Chem..

[19] Soto-Hernández M., García-Mateos R., Palma-Tenango M. (2019). Plant Physiological Aspects of Phenolic Compounds.

[20] Marchiosi R., Dos Santos W.D., Constantin R.P., De Lima R.B., Soares A.R., Finger-Teixeira A., Mota T.R., de Oliveira D.M., Foletto-Felipe M.D.P., Abrahão J. (2020). Biosynthesis and metabolic actions of simple phenolic acids in plants. Phytochem. Rev..

[21] Kumar N., Goel N. (2019). Phenolic acids: Natural versatile molecules with promising therapeutic applications. Biotechnol. Rep..

[22] Robbins R.J. (2003). Phenolic Acids in Foods: An Overview of Analytical Methodology. J. Agric. Food Chem..

[23] Singh B.N., Singh R., Prakash D., Sarma B., Singh H. (2009). Antioxidant and anti-quorum sensing activities of green pod of *Acacia nilotica* L. Food Chem. Toxicol..

[24] Subhan N., Burrows G.E., Kerr P.G., Obied H.K. (2018). Phytochemistry, ethnomedicine, and pharmacology of Acacia. Stud. Nat. Prod. Chem..

[25] Landete J.M. (2011). Ellagitannins, ellagic acid and their derived metabolites: A review about source, metabolism, functions and health. Food Res. Int..

[26] Kähkönen M.P., Hopia A.I., Heinonen M. (2001). Berry Phenolics and Their Antioxidant Activity. J. Agric. Food Chem..

[27] Muthukumaran S., Tranchant C., Shi J., Ye X., Xue S.J. (2017). Ellagic acid in strawberry (*Fragaria* spp.): Biological, technological, stability, and human health aspects. Food Qual. Saf..

[28] Pinto M.D.S., Lajolo F.M., Genovese M.I. (2008). Bioactive compounds and quantification of total ellagic acid in strawberries (*Fragaria x ananassa* Duch.). Food Chem..

[29] Vazirian M., Khanavi M., Amanzadeh Y., Hajimehdipoor H. (2011). Quantification of Gallic Acidin Fruits of Three Medicinal Plants. Iran. J. Pharm. Res. IJPR.

[30] Mirunalini S., Krishnaveni M. (2010). Therapeutic potential of Phyllanthus emblica (amla): The ayurvedic wonder. J. Basic Clin. Physiol. Pharmacol..

[31] Khan A.K., Rashid R., Fatima N., Mahmood S., Mir S., Khan S., Jabeen N., Murtaza G. (2015). Pharmacological activities of protocatechuic acid. Acta Pol. Pharm..

[32] Kim J.K., Park S.U. (2019). A recent overview on the biological and pharmacological activities of ferulic acid. EXCLI J..

[33] Manjunatha B.K. (2006). Antibacterial activity of *Pterocarpus santalinus*. Indian J. Pharm. Sci..

[34] Manuja R., Sachdeva S., Jain A., Chaudhary J. (2013). A comprehensive review on biological activities of p-hydroxy benzoic acid and its derivatives. Int. J. Pharm. Sci. Rev. Res..

[35] Chong K., Zuraini Z., Sasidharan S., Devi P.K., Latha L.Y., Ramanathan S. (2008). Antimicrobial activity of *Elaeis guineensis* leaf. Pharmacologyonline.

[36] Heinonen M.I. (1990). Carotenoids and provitamin A activity of carrot (*Daucus carota* L.) cultivars. J. Agric. Food Chem..

[37] Abedi F., Razavi B.M., Hosseinzadeh H. (2019). A review on gentisic acid as a plant derived phenolic acid and metabolite of aspirin: Comprehensive pharmacology, toxicology, and some pharmaceutical aspects. Phytother. Res..

[38] Holobiuc I., Blîndu R. (2008). In vitro culture of the protected rare species *Gentiana lutea* L. for conservative purpose. Contrib. Bot..

[39] Momčilović I., Grubiršić D., Nešković M., Bajaj Y.P.S. (2001). Transgenic *Gentiana* species (Gentian). Transgenic Crops III.

[40] Luís Â., Neiva D., Pereira H., Gominho J., Domingues F., Duarte A.P. (2014). Stumps of Eucalyptus globulus as a Source of Antioxidant and Antimicrobial Polyphenols. Molecules.

[41] Rababah T.M., Ereifej K.I., Esoh R.B., Al-u'datt M.H., Alrababah M.A., Yang W. (2011). Antioxidant activities, total phenolics and HPLC analyses of the phenolic compounds of extracts from common Mediterranean plants. Nat. Prod. Res..

[42] Kurata R., Adachi M., Yamakawa O., Yoshimoto M. (2006). Growth Suppression of Human Cancer Cells by Polyphenolics from Sweetpotato (*Ipomoea batatas* L.) Leaves. J. Agric. Food Chem..

[43] Ashrafizadeh M., Zarrabi A., Hashemi F., Moghadam E.R., Hashemi F., Entezari M., Najafi M. (2020). Curcumin in cancer therapy: A novel adjunct for combination chemotherapy with paclitaxel and alleviation of its adverse effects. Life Sci..

[44] Vogt P.K., Kang S., Elsliger M.-A., Gymnopoulos M. (2007). Cancer-specific mutations in phosphatidylinositol 3-kinase. Trends Biochem. Sci..

[45] Halilovic E., She Q.-B., Ye Q., Pagliarini R., Sellers W.R., Solit D.B., Rosen N. (2010). PIK3CA Mutation Uncouples Tumor Growth and Cyclin D1 Regulation from MEK/ERK and Mutant KRAS Signaling. Cancer Res..

[46] Rosa L.d.S., Silva N.J.A., Soares N.C.P., Monteiro M.C., Teodoro A.J. (2016). Anticancer properties of phenolic acids in colon cancer—A review. J. Nutr. Food Sci..

[47] Chander M. (2018). Anticancer efficacy of some plant Phenolics—A recent scenario. Int. J. Curr. Microbiol. Appl. Sci..

[48] Choi J., Jiang X., Jeong J.B., Lee S.-H. (2014). Anticancer Activity of Protocatechualdehyde in Human Breast Cancer Cells. J. Med. Food.

[49] Narayanan B.A., Geoffroy O., Willingham M.C., Re G.G., Nixon D.W. (1999). p53/p21 (WAF1/CIP1) expression and its possible role in G1 arrest and apoptosis in ellagic acid treated cancer cells. Cancer Lett..

[50] Forester S.C., Choy Y.Y., Waterhouse A.L., Oteiza P.I. (2012). The anthocyanin metabolites gallic acid, 3-*O*-methylgallic acid, and 2,4,6-trihydroxybenzaldehyde decrease human colon cancer cell viability by regulating pro-oncogenic signals. Mol. Carcinog..

[51] Chung Y.-C., Lu L.-C., Tsai M.-H., Chen Y.-J., Chen Y.-Y., Yao S.-P., Hsu C.-P. (2013). The Inhibitory Effect of Ellagic Acid on Cell Growth of Ovarian Carcinoma Cells. Evid.-Based Complement. Altern. Med..

[52] Janicke B., Önning G., Oredsson S.M. (2005). Differential Effects of Ferulic Acid and *p*-Coumaric Acid on S Phase Distribution and Length of S Phase in the Human Colonic Cell Line Caco-2. J. Agric. Food Chem..

[53] Bouzaiene N.N., Jaziri S.K., Kovacic H., Chekir-Ghedira L., Ghedira K., Luis J. (2015). The effects of caffeic, coumaric and ferulic acids on proliferation, superoxide production, adhesion and migration of human tumor cells in vitro. Eur. J. Pharmacol..

[54] Lin H.P., Jiang S.S., Chuu C.P. (2012). Caffeic acid phenethyl ester causes p21Cip1 induction, Akt signaling reduction, and growth inhibition in PC-3 human prostate cancer cells. PLoS ONE.

[55] Chiang E., Tsai S.-Y., Kuo Y.-H., Pai M.-H., Chiu H.-L., Rodriguez R.L., Tang F.-Y. (2014). Caffeic Acid Derivatives Inhibit the Growth of Colon Cancer: Involvement of the PI3-K/Akt and AMPK Signaling Pathways. PLoS ONE.

[56] Cheng C.-Y., Ho T.-Y., Lee E.-J., Su S.-Y., Tang N.-Y., Hsieh C.-L. (2008). Ferulic Acid Reduces Cerebral Infarct Through Its Antioxidative and Anti-Inflammatory Effects Following Transient Focal Cerebral Ischemia in Rats. Am. J. Chin. Med..

[57] Min S.-W., Ryu S.-N., Kim D.-H. (2010). Anti-inflammatory effects of black rice, cyanidin-3-O-β-d-glycoside, and its metabolites, cyanidin and protocatechuic acid. Int. Immunopharmacol..

[58] Xian Y.-F., Hu Z., Ip S.-P., Chen J.-N., Su Z.-R., Lai X.-P., Lin Z.-X. (2018). Comparison of the anti-inflammatory effects of Sinapis alba and Brassica juncea in mouse models of inflammation. Phytomedicine.

[59] Jaganathan S.K. (2012). Growth Inhibition by Caffeic Acid, One of the Phenolic Constituents of Honey, in HCT 15 Colon Cancer Cells. Sci. World J..

[60] Gao K., Xu A., Krul C., Venema K., Liu Y., Niu Y., Lu J., Bensoussan L., Seeram N.P., Heber D. (2006). Of the Major Phenolic Acids Formed during Human Microbial Fermentation of Tea, Citrus, and Soy Flavonoid Supplements, Only 3,4-Dihydroxyphenylacetic Acid Has Antiproliferative Activity. J. Nutr..

[61] Kong C.K., Lam W., Chiu L.C., Ooi V.E., Sun S.S., Wong Y.-S. (2009). A rice bran polyphenol, cycloartenyl ferulate, elicits apoptosis in human colorectal adenocarcinoma SW480 and sensitizes metastatic SW620 cells to TRAIL-induced apoptosis. Biochem. Pharmacol..

[62] Lin L.Z., Harnly J.M. (2010). Identification of the phenolic components of chrysanthemum flower (Chrysanthemum morifolium Ramat). Food Chem..

[63] Mihanfar A., Darband S.G., Sadighparvar S., Kaviani M., Mirza-Aghazadeh-Attari M., Yousefi B., Majidinia M. (2021). In vitro and in vivo anticancer effects of syringic acid on colorectal cancer: Possible mechanistic view. Chem. Interact..

[64] Papoutsi Z., Kassi E., Tsiapara A., Fokialakis N., Chrousos G.P., Moutsatsou P. (2005). Evaluation of Estrogenic/Antiestrogenic Activity of Ellagic Acid via the Estrogen Receptor Subtypes ERα and ERβ. J. Agric. Food Chem..

[65] Kalezic A., Macanovic B., Garalejic E., Korac A., Otasevic V., Korac B. (2018). Level of NO/nitrite and 3-nitrotyrosine in seminal plasma of infertile men: Correlation with sperm number, motility and morphology. Chem. Interact..

[66] Pitchakarn P., Chewonarin T., Ogawa K., Suzuki S., Asamoto M., Takahashi S., Shirai T., Limtrakul P. (2013). Ellagic Acid Inhibits Migration and Invasion by Prostate Cancer Cell Lines. Asian Pac. J. Cancer Prev..

[67] Kaur M., Velmurugan B., Rajamanickam S., Agarwal R., Agarwal C. (2009). Gallic Acid, an Active Constituent of Grape Seed Extract, Exhibits Anti-proliferative, Pro-apoptotic and Anti-tumorigenic Effects Against Prostate Carcinoma Xenograft Growth in Nude Mice. Pharm. Res..

[68] Gong J., Zhou S., Yang S. (2019). Vanillic Acid Suppresses HIF-1α Expression via Inhibition of mTOR/p70S6K/4E-BP1 and Raf/MEK/ERK Pathways in Human Colon Cancer HCT116 Cells. Int. J. Mol. Sci..

[69] Eroğlu C., Avcı E., Vural H., Kurar E. (2018). Anticancer mechanism of Sinapic acid in PC-3 and LNCaP human prostate cancer cell lines. Gene.

[70] Hori J.I., Zamboni D.S., Carrão D.B., Goldman G.H., Berretta A.A. (2013). The Inhibition of Inflammasome by Brazilian Propolis (EPP-AF). Evid.-Based Complement. Altern. Med..

[71] Rabelo T.K., Guimarães A.G., Oliveira M.A., Gasparotto J., Serafini M.R., de Souza Araújo A.A., Quintans-Júnior L.J., Moreira J.C.F., Gelain D.P. (2016). Shikimic acid inhibits LPS-induced cellular pro-inflammatory cytokines and attenuates mechanical hyperalgesia in mice. Int. Immunopharmacol..

[72] Da Cunha F.M., Duma D., Assreuy J., Buzzi F.C., Niero R., Campos M.M., Calixto J.B. (2004). Caffeic Acid Derivatives: In Vitro and In Vivo Anti-inflammatory Properties. Free Radic. Res..

[73] Pragasam S.J., Venkatesan V., Rasool M. (2012). Immunomodulatory and Anti-inflammatory Effect of p-Coumaric Acid, a Common Dietary Polyphenol on Experimental Inflammation in Rats. Inflammation.

[74] Wang D., Wei X., Yan X., Jin T., Ling W. (2010). Protocatechuic Acid, a Metabolite of Anthocyanins, Inhibits Monocyte Adhesion and Reduces Atherosclerosis in Apolipoprotein E-Deficient Mice. J. Agric. Food Chem..

[75] Leifert W.R., Abeywardena M.Y. (2008). Cardioprotective actions of grape polyphenols. Nutr. Res..

[76] Taofiq O., González-Paramás A.M., Barreiro M.F., Ferreira I.C. (2017). Hydroxycinnamic acids and their derivatives: Cosmeceutical significance, challenges and future perspectives, a review. Molecules.

[77] Iriti M., Faoro F. (2009). Bioactivity of Grape Chemicals for Human Health. Nat. Prod. Commun..

[78] Fuhrman B., Volkova N., Coleman R., Aviram M. (2005). Grape Powder Polyphenols Attenuate Atherosclerosis Development in Apolipoprotein E Deficient (E0) Mice and Reduce Macrophage Atherogenicity. J. Nutr..

[79] Frederiksen H., Mortensen A., Schrøder M., Frandsen H., Bysted A., Knuthsen P., Rasmussen S.E. (2007). Effects of red grape skin and seed extract supplementation on atherosclerosis in Watanabe heritable hyperlipidemic rabbits. Mol. Nutr. Food Res..

[80] Saibabu V., Fatima Z., Khan L.A., Hameed S. (2015). Therapeutic Potential of Dietary Phenolic Acids. Adv. Pharmacol. Sci..

[81] Mouhamed D., Ezzaher A., Gaha L., Douki W., Najjar M.F. (2013). In vitro effects of salicylic acid on plasma paraoxonase 1 activity. J. Drug Metab. Toxicol..

[82] Zhou L., Yu X., Meng Q., Li H., Niu C., Jiang Y., Cai Y., Li M., Li Q., An C. (2013). Resistin reduces mitochondria and induces hepatic steatosis in mice by the protein kinase C/protein kinase G/p65/PPAR gamma coactivator 1 alpha pathway. Hepatology.

[83] Sun S., Kee H.J., Jin L., Ryu Y., Choi S.Y., Kim G.R., Jeong M.H. (2018). Gentisic acid attenuates pressure overload-induced cardiac hypertrophy and fibrosis in mice through inhibition of the ERK 1/2 pathway. J. Cell. Mol. Med..

[84] Altinoz M.A., Elmaci I., Cengiz S., Emekli-Alturfan E., Ozpinar A. (2018). From epidemiology to treatment: Aspirin’s prevention of brain and breast-cancer and cardioprotection may associate with its metabolite gentisic acid. Chem.-Biol. Interact..

[85] Yogeeta S.K., Gnanapragasam A., Kumar S.S., Subhashini R., Sathivel A., Devaki T. (2006). Synergistic interactions of Ferulic acid with Ascorbic acid: Its cardioprotective role during isoproterenol induced myocardial infarction in rats. Mol. Cell. Biochem..

[86] Sarr M., Chataigneau M., Martins S., Schott C., El Bedoui J., Oak M.-H., Muller B., Chataigneau T., Schini-Kerth V.B. (2006). Red wine polyphenols prevent angiotensin II-induced hypertension and endothelial dysfunction in rats: Role of NADPH oxidase. Cardiovasc. Res..

[87] Lecour S., Lamont K.T. (2011). Natural polyphenols and cardioprotection. Mini Rev. Med. Chem..

[88] Opie L.H., Commerford P.J., Gersh B.J., Pfeffer M.A. (2006). Controversies in ventricular remodelling. Lancet.

[89] Tan J., Ma Z., Han L., Du R., Zhao L., Wei X., Hou D., Johnstone B.H., Farlow M.R., Du Y. (2005). Caffeic acid phenethyl ester possesses potent cardioprotective effects in a rabbit model of acute myocardial ischemia-reperfusion injury. Am. J. Physiol. Circ. Physiol..

[90] Aswar U., Mahajan U., Kandhare A., Aswar M. (2019). Ferulic acid ameliorates doxorubicin-induced cardiac toxicity in rats. Naunyn-Schmiedeberg’s Arch. Pharmacol..

[91] Ding S.-K., Wang L.-X., Guo L.-S., Luo P., Du J.-J., Zhao Z.-L., Wang G.-G. (2017). Syringic acid inhibits apoptosis pathways via downregulation of p38MAPK and JNK signaling pathways in H9c2 cardiomyocytes following hypoxia/reoxygenation injury. Mol. Med. Rep..

[92] Shaik A.H., Al Omar S.Y., Mohammad A., Kodidhela L.D. (2020). Combined cardio-protective ability of syringic acid and resveratrol against isoproterenol induced cardio-toxicity in rats via attenuating NF-kB and TNF-α pathways. Sci. Rep..

[93] Roy A.J., Prince P.S.M. (2013). Preventive effects of p-coumaric acid on cardiac hypertrophy and alterations in electrocardiogram, lipids, and lipoproteins in experimentally induced myocardial infarcted rats. Food Chem. Toxicol..

[94] Tang X.-L., Liu J.-X., Dong W., Li P., Li L., Lin C.-R., Zheng Y.-Q., Cong W.-H., Hou J.-C. (2014). The Cardioprotective Effect of Protocatechuic Acid on Myocardial Ischemia/Reperfusion Injury. J. Pharmacol. Sci..

[95] Semaming Y., Kumfu S., Pannangpetch P., Chattipakorn S.C., Chattipakorn N. (2014). Protocatechuic acid exerts a cardioprotective effect in type 1 diabetic rats. J. Endocrinol..

